# Intensive care and disaster medicine: the role of a compendium

**DOI:** 10.1186/cc12217

**Published:** 2013-03-19

**Authors:** Y Haraguchi, Y Tomoyasu, H Nishi, M Hoshino, T Tsubata, M Sakai, E Hoshino

**Affiliations:** 1 Disaster Medicine Compendium Team, Japan, Tokyo, Japan; 2 Keiyo Hospital, Tokyo, Japan

## Introduction

There were several catastrophes in the last decades. The make-up of systematic measuring and life-saving medical systems, including intensive care, is thought to be an urgent and essential issue. Our efforts for establishing a disaster medicine and education system are presented.

## Methods

Mega-disasters or catastrophes are researched basically on actual medical experience; that is, the 9/11 attack in 2001, Hurricane Katrina in 2005, Indian Ocean earthquake with tsunami in 2004, the Chernobyl incident in 1986 as well as the Higashinihon earthquakes with tsunami, which was followed by the severest degree of nuclear disaster in 2011.

## Results

Our research indicated that disaster medicine should be established systematically or it is necessary to compile a compendium of disaster medicine from a broad perspective or from a bird's-eye and long-term view. The Japanese version was tentatively completed with 22 volumes as of the financial year 2005, of which nearly three-quarters are written in Japanese. Although this worked partly during the above-shown catastrophe in Japan 2011, several problems are left to be solved; that is, the insufficient operation system of the Japan DMAT or Disaster Medical Assistant Team that seemed to have caused a large number of preventable deaths.

## Conclusion

The large number of casualties during a major disaster is a global problem, even in the developed countries. When the role of the intensivist is reviewed, many roles were verified to be important; that is, as a leader of a medical team or triage officer as well as a professional in the field of specific intensive care. However, there are many problems to be solved in the fields of disaster medicine. In order to solve the diversification or the various medical problems, it is necessary to compile or systematize a disaster medicine of the world version. The concept of the compendium and our process of trial are shown in relation to intensive care.

